# Novel Biocement/Honey Composites for Bone Regenerative Medicine

**DOI:** 10.3390/jfb14090457

**Published:** 2023-09-04

**Authors:** Lubomir Medvecky, Maria Giretova, Radoslava Stulajterova, Tibor Sopcak, Pavlina Jevinova, Lenka Luptakova

**Affiliations:** 1 Division of Functional and Hybrid Systems, Institute of Materials Research of SAS, Watsonova 47, 040 01 Kosice, Slovakia; lmedvecky@saske.sk (L.M.); mgiretova@saske.sk (M.G.); tsopcak@saske.sk (T.S.); 2 Department of Food Hygiene, Technology and Safety, University of Veterinary Medicine and Pharmacy, Komenskeho 73, 041 81 Kosice, Slovakia; pavlina.jevinova@uvlf.sk; 3 Department of Biology and Physiology, University of Veterinary Medicine and Pharmacy in Kosice, Komenskeho 73, 041 81 Kosice, Slovakia; lenka.luptakova@uvlf.sk

**Keywords:** biocement, honey, antimicrobial properties, antioxidant properties, osteogenic potential

## Abstract

New biocements based on a powdered mixture of calcium phosphate/monetite (TTCPM) modified with the addition of honey were prepared by mixing the powder and honey liquid components at a non-cytotoxic concentration of honey (up to 10% (*w*/*v*)). The setting process of the cements was not affected by the addition of honey, and the setting time of ~4 min corresponded to the fast setting calcium phosphate cements (CPCs). The cement powder mixture was completely transformed into calcium-deficient nanohydroxyapatite after 24 h of hardening in a simulated body fluid, and the columnar growth of long, needle-like nanohydroxyapatite particles around the original calcium phosphate particles was observed in the honey cements. The compressive strength of the honey cements was reduced with the content of honey in the cement. Comparable antibacterial activities were found for the cements with honey solutions on *Escherichia coli*, but very low antibacterial activities were found for *Staphylococcus aureus* for all the cements. The enhanced antioxidant inhibitory activity of the composite extracts was verified. In vitro cytotoxicity testing verified the non-cytotoxic nature of the honey cement extracts, and the addition of honey promoted alkaline phosphatase activity, calcium deposit production, and the upregulation of osteogenic genes (osteopontin, osteocalcin, and osteonectin) by mesenchymal stem cells, demonstrating the positive synergistic effect of honey and CPCs on the bioactivity of cements that could be promising therapeutic candidates for the repair of bone defects.

## 1. Introduction

Large, critical bone defects (>2 cm in diameter in humans) cannot be spontaneously repaired through reparative mechanisms and need help from the outside in the form of grafts or scaffolds with the appropriate biological and mechanical properties (preferably supplemented with cells and growth factors) [1,2]. Nearly 2 million bone grafts (including autografts, allografts, and xenografts) are performed worldwide annually. Autografts are still considered as the gold standard, but can cause bleeding, hematomas, and pain due to the invasiveness of the surgical technique; on the other hand, allografts and xenografts may cause a risk of disease transmission or immunological reaction. However, no scaffold is considered the best candidate for healing large bone defects, as bone tissue healing is affected by the patient’s age, lifestyle, and overall health [3]. Calcium phosphate cements (CPCs) belong to an important class of biomaterials which are characterized by excellent biocompatibility and osteoconductivity, non-cytotoxicity, and bioresorption, which predetermines them for possible use in the human body for bone repair and replacement [4]. CPCs generally consist of a powder and a liquid component that are mixed together to form a paste which can optimally fit the bone defect regardless of its shape [5]. This one-of-a-kind CPC is based on a mixture of basic tetracalcium phosphate (TTCP) and acidic brushite or monetite (DCPA) components which are transformed during cement setting into the nanocrystalline calcium-deficient hydroxyapatite (CDHA). CDHA is chemically and structurally similar to the mineral component of the mammalian bone. It has been shown that fast-setting tetracalcium phosphate/monetite (TTCPM) biocements, especially those with a modified composition, can promote the osteogenic differentiation of mesenchymal stem cells into osteoblasts [6]. On the other hand, adding a number of heavy metal ions or increasing the pH during the setting process improves the antimicrobial activity of the CPCs [7,8].

Honey is one of the most complex natural foods and contains more than 200 substances; its composition strongly depends on its botanical and geographical origins [9]. Honey has been found to stimulate the healing of wounds and burns and is also effective in the treatment of infected wounds [10]. The main constituents of honey are sugars (~80%), water (20%), and other ingredients such as proteins (enzymes), organic acids, vitamins, minerals, pigments, phenolic compounds, etc. Phenolic compounds are responsible for honey’s diverse color and flavor, and they induce antioxidant and antimicrobial properties [11]. The acidity of honey (pH 3.2–4.5) increases its antibacterial properties since the appropriate pH for the growth of most bacterial strains is 6.5–7.5. The inhibition of microbial growth caused by honey is attributable to H_2_O_2_ and various non-peroxide substances (including phenolic compounds, organic acids, and flavonoids) and important supporting factors with a low water content (high osmolarity), a high concentration of sugars, the presence of bee defensin-1, and methylglyoxal [12,13,14]. In addition, the positive effects of honey on bone metabolism, osteoporosis, and bone fracture healing have been demonstrated [15,16]; however, despite these facts, only a few papers focusing on analyzing the effect of honey addition on the properties of bioactive cements have been published so far.

**Scheme 1:**
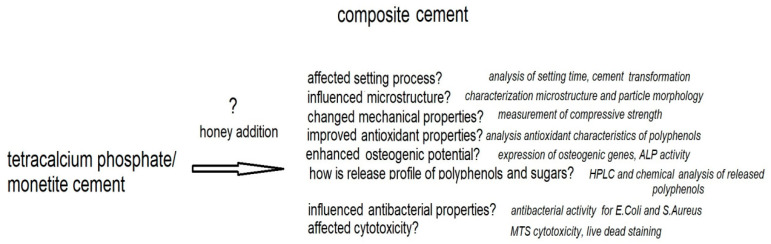
Schematic diagram showing the questions that are addressed in this work.

The aim of this work was to evaluate the properties of a new composite cement based on a TTCP/monetitic cement mixture with the addition of honey, where calcium phosphates support the bioactivity of the cells through calcium and phosphate ions and, on the other hand, honey can influence the behavior of the cells in terms of their osteogenic activity, as well as the antibacterial and antioxidant properties of cement due to the presence of specific components of honey such as polyphenols or flavonoids. In this paper, the effect of honey addition (two kinds of honey) on the setting process, microstructure, mechanical and antibacterial properties, in vitro cytotoxicity of cements, and osteogenic activity of cells cultured in cement extracts is elucidated (Scheme 1).

## 2. Materials and Methods

### 2.1. Preparation of the Cement Powder Mixture

The tetracalcium phosphate/monetite cement powder mixture (Ca/P mole ratio = 1.67) was synthesized in situ via mixing tetracalcium phosphate (TTCP) with a diluted ethanolic solution (80% ethanol) of the orthophosphoric acid (86% analytical grade, Merck, Darmstadt, Germany) in a planetary ball mill (Retsch, 600 rpm, agate balls and vessel for 30 min at room temperature). The final powder mixture was composed of TTCP and monetite (DCPA) in an equimolar ratio. Tetracalcium phosphate was synthesized by a solid state reaction between calcium carbonate (CaCO_3_, analytical grade, Sigma Aldrich, Saint Louis, MO, USA) and calcium hydrogen phosphate anhydrous (monetite, DCPA) (CaHPO_4_ (Ph.Eur., Fluka) at 1450 °C for 5 h. The synthesized TTCP was milled in a planetary ball mill (Retsch PM100, 350 rpm) for 1 h prior to further treatment.

### 2.2. Formation of Cement Pastes and Characterization of Honeys

#### 2.2.1. Preparation of Cement Pastes

The liquid component for the preparation of cement pastes was a 2% solution of NaH_2_PO_4_ (analytical grade, Sigma-Aldrich, Steinheim, Germany), and the honeys were also dissolved in this solution. The two kinds of medical honey that were selected for use in our study were manuka-based honey (Activon^®^, medical grade, Advancis medical, Nottinghamshire, UK) and chestnut honey (Vivamel^®^, Tosama, medical grade, Domžale, Slovenija). The contents of the two types of honey in the liquid component were 5 and 10% (*w*/*v*); the powder to liquid (P/L) ratio was 2. The cements prepared with 5 and 10% (*w*/*v*) honey in the liquid component were designated as M5, V5 or M10, and V10, where M and V represent the manuka-based and chestnut honey, respectively.

#### 2.2.2. Analysis of Proteins in Honeys

The total protein content in the honeys was determined using Coomasie blue method [17] and bovine serum albumin as a standard; the molecular mass protein profile was analyzed through using SDS PAGE gel electrophoresis on polyacrylamide gels (NuPAGE, 4–12% Bis-Tris gel, 1 mm, Invitrogen, Carlsbad, CA, USA) with NuPAGE MOPS running buffer in X’CellSurelock MiniCell (200 V, 100 mA for 45 min) at native conditions using NuPAGE LDS sample buffer according to the manufacturer’s conditions. Silver staining (SilverXpress, Invitrogen, Carlsbad, CA, USA) was used for the detection of proteins in the gel, and the molecular mass of proteins was standardized by using the Novex sharp unstained protein standard kit (Invitrogen, Carlsbad, CA, USA). Prior to analysis, the proteins in the dissolved honeys (200 mg/1.5 mL of final solution) were precipitated with saturated (NH_4_)_2_SO_4_ solution and kept at 4 °C for 12 h, centrifuged at 10,000 rpm (HETTICH, Mikro220R, Tuttlingen, Germany), and recovered in distilled water. Imaging analysis was used to facilitate a profile analysis of the proteins in lanes (ImageJ 8 software).

#### 2.2.3. Determination of Total Phenols, Flavonoids, and Sugars in Honeys and Cement Extracts during Release to SBF

The total amount of polyphenols in honeys and cement extracts during soaking in SBF was determined by using Folin–Ciocalteu reagent (FC) [18]. Briefly, 100 μL FC was added to 100 μL of 10% (*w*/*v*) honey aqueous solution, and 200 µL of 5% (*w*/*v*) Na_2_CO_3_ solution was admixed to mixture. After 30 min, the absorbance of the reaction mixture was measured at 750 nm using a UV-VIS spectrophotometer (SHIMADZU, UV-VIS 1800, Kyoto, Japan). Gallic acid was selected as the standard for calibration, and the results were expressed as μg of GAE/g of honey.

The flavonoid content in the honeys was analyzed using a method based on the formation of complexes with AlCl_3_ [19]. A total of 1 mL of 12% (*w*/*v*) honey was mixed with 300 μL of NaNO_2_ solution (5% *w*/*v*), and after 5 min, 10% (*w*/*v*) AlCl_3_ solution was added to reaction mixture. As a final step, 2 mL of 10% (*w*/*v*) Na_2_CO_3_ solution was added. The reactants in the solution were mixed together, and the flavonoid content was calculated from the calibration curve using quercetin (QUE) as the standard. The results were expressed as μg of QUE/g of honey.

The profile of the phenolic compounds in the honeys and cement extracts after soaking in 0.9% NaCl solution was determined using HPLC chromatography. HPLC analysis was performed using a liquid chromatograph (Watrex) equipped with a multichannel UV-VIS detector (SYKAM, S3240, Eresing, Germany), binary pumps (DeltaChrom), and online vacuum degasser (Watrex), Nucleosil 100-5C18, 4.6 × 250 mm column (Supelco, Dorset, UK) at a flow rate of 1 mL min^−1^, and the results were evaluated using Clarity Datastation Software. Solvent gradient was performed by varying the proportion of solvent A (0.5% formic acid in water (*v*/*v*)) to solvent B (methanol) as follows: initial 95% A; 0–20 min, 95 −30% A; 20–25 min, 30–10% A; 25–30 min, 10–5% A. The total running time and post-running time were 35 and 10 min, respectively. The injected volume of samples and standards was 20 μL. The four wavelengths at 250, 270, 320, and 370 nm were selected for detection of polyphenols.

The honey cement extracts were prepared via soaking the cement pastes composed of 1 g of powder cement mixture and 500 μL of liquid component with 10% (*w*/*v*) honey content (V10, M10)) in 10 mL of pure 0.9% NaCl solution to eliminate any artifacts in the HPLC chromatograms from possible impurities in used organic additives. After the selected soaking times had elapsed (3, 24, 48, 120, and 168 h), the solution was filtered through a PVDF membrane filter (Millipore, pore size 0.22 μm), and polyphenols were extracted from the soaking solution via solid-phase extraction (SPE cartridge, Bond Elut C18, 100 mg, Agilent Technologies, Santa Clara, CA, USA) according to the manufacturer’s instructions. The final ~1 mL of methanolic solution containing the polyphenols was lyophilized, and the polyphenols were redissolved in 200 μL of methanol. For a more accurate analysis, the recovery of individual selected polyphenolic compounds after SPE extraction was determined using 1 and 10 μg of polyphenol/10 mL solution under the same conditions used for the extraction of compounds from soaking solutions, and the results from the HPLC analysis were corrected by the corresponding degree of recovery. The released amount of selected polyphenolic acids and flavonoids from cements during soaking in 0.9% NaCl solution was expressed relative to the total content of polyphenols in cement pastes.

In addition, filtrates containing the separated sugar solution after the extraction of polyphenols by SPE were used to analyze the sugar content in the honeys and determine the sugar content in the cement extracts during soaking. HPLC chromatography (SupelcoSIL LC-NH2, column: 4.6 × 250 mm, size: 5 μm, and isocratic analysis conditions with mobile phase water/acetonitrile (25:75), RI detector (RI200, Schambeck GmbH, Bad Honnef, Germany), and 1 mL/min flow rate) was used to determine the sugar content in the samples. The amount of sugar released from the cements during soaking in 0.9% NaCl solution was expressed relative to the total content of individual sugars in the cement pastes.

#### 2.2.4. Free Radical Scavenging Activity and Total Antioxidant Content

The free radical scavenging activity (FRSA) of the honeys was characterized by DPPH^•^ (1,1-diphenyl-2-picrylhydrazyl) and ABTS^•+^ (2,2′-azinobis-3-ethylbenzothiazoline-6-sulfuric acid) radical scavenging assays, whereas the FRSA of the cement extracts after soaking in SBF at selected time periods (1, 2, 5, and 7 days) was determined using only the ABTS^•+^ assay. This method was applied because the reactive ABTS^•+^ radical and the final products are soluble in aqueous solution (SBF), in which the release of active substances (e.g., polyphenols) was measured, and the experimental conditions were more similar to the physiological environment. On the other hand, the DPPH^•^ assay, which is soluble in organic solvents, is appropriate for the analysis of lipophilic antioxidants (insoluble in water solutions) [20,21]. However, it is unlikely to assume the high activity of the water-insoluble substances without some degree of conversion by metabolic processes.

Briefly, 500 μL of 2.5% (*w*/*v*) honey methanolic solution was mixed with 1 mL of 0.06 mM DPPH methanolic solution, and the absorbance of solution was measured at 517 nm using a UV-VIS spectrophotometer (UV-VIS 1800, Shimadzu, Japan) after 15 min of reaction. Regarding the ABTS^•+^ method, 100 µL of 2.5% (*w*/*v*) of honey solution or cement extract was reacted with 1 mL of ABTS^•+^ reagent, and absorbance at 734 nm was measured after 15 min of reaction. The ABTS^•+^ reagent was prepared by diluting the origin ABTS^•+^ stock solution with phosphate-buffered saline (PBS). The reactive ABTS^•+^ radical was synthesized by reacting a 7 mM ABTS solution (5 mL) with 140 mM K_2_S_2_O_8_ (88 μL) in a molar ratio of 1:0.5 at room temperature for 12 h [21].

The total antioxidant content (TAC) in honeys and cement extracts was determined using the ABTS method, wherein ascorbic acid (0–10 μg/mL) was used as the standard for calibration, and the results were expressed in ascorbic acid antioxidant content equivalents (AAE) (mg of ascorbic acid per gram of honey).

The cement extracts intended for FRSA and TAC analysis were obtained from cement pastes composed of 2.5 g of cement powder mixture and 1.25 mL of 10% (*w*/*v*) honey dissolved in 2% (*w*/*v*) NaH_2_PO_4_ solution during soaking in 10 mL SBF for 1, 2, 5 and 7 days.

FRSA was calculated according following to equation (Equation (1)):

ABTS (or DPPH) FRSA (%) = 100 × (Abs_C_ − Abs_s_)/Abs_C_ (1)

where Abs_C_—absorbance of control, and Abs_s_—absorbance of sample.

#### 2.2.5. Characterization of Microstructures, Setting Time, and Mechanical Properties

The compressive strength (CS) of the cements was measured on a universal testing machine (5 kN load cell, LR5K Plus, Lloyd Instruments Ltd., West Sussex, UK) at a crosshead speed of 1 mm/min (mean + standard deviation, *n* = 4). The measurement samples were prepared from the cement pastes via packing in stainless cylindrical form (6 mm D × 12 mm H), hardening in 100% humidity at 37 °C for 10 min, and soaking in simulate body fluid (SBF) at 37 °C for 1 week. The phase compositions of the samples were characterized using X-ray diffraction analysis (Philips X PertPro, Malvern Panalytical B.V., Eindhoven, the Netherlands, Cu Kα radiation 50 mA, 2Θ range 20–40°) and FTIR spectroscopy (IRAffinity1, Shimadzu, Kyoto, Japan, 400 mg KBr + 1 mg sample).

The final cement microstructures of the fractured surfaces were observed via field-emission scanning electron microscopy (JEOL FE SEM JSM-7000F, Tokyo, Japan) after coating with carbon. The morphology of the calcium phosphate particles in the cements was analyzed using transmission electron microscopy (JEOL JEM 2100F, Tokyo, Japan).

The final setting times (ST) of the cement pastes were evaluated using the tip (1 mm diameter) of a Vicat needle with a 400 g load (according to ISO standard 1566), which failed to make a perceptible circular indentation on the surface of the cement.

The changes in the concentration of the calcium and phosphate ions released from the cements while the cement pellets were soaked in SBF solution at 37 °C (400 mg/15 mL of solution, polypropylene tube, shaken) were analyzed via ICP (Horiba Activa, Longjumeau, France) after filtration with a membrane filter (PVDF, 45 µm pore size, Millipore, Darmstadt, Germany) at selected soaking times of 0, 2, 4, 6, 24, 48 and 168 h.

### 2.3. Preparation of Cement Extracts and In Vitro Cytotoxicity Testing of Extracts

To evaluate contact cytotoxicity, the cement samples (Ø 6 mm, 1 mm in thickness) were sterilized under UV light in a laminar box for 30 min on each side of the samples and placed on a 48–well culture plate (TPP, Trasadingen, Switzerland). Mouse preosteoblastic MC3T3E1 Subclone 4 cells (ATCC CRL-2593, Manassas, VA, USA) were enzymatically released from culture flasks, and the cell suspension was adjusted to a density of 5.0 × 10^4^ cells/mL. Additionally, 2.0 × 10^4^ cells in 400 μL of culture medium consisting of EMEM (Eagle’s Minimum Essential Medium), 10% FBS, 1% antibiotic solution (all from Sigma) were added to each tested sample. The density, distribution, and morphology of the cells were assessed after 2 days of cultivation via live/dead fluorescent staining (fluorescein diacetate FDA/propidium iodide PI) using an inverted optical fluorescence microscope (Leica DM IL LED, blue filter).

To determine the cytotoxicity of the extracts, MC3T3E1 cells were resuspended in culture medium, and the cell density was adjusted to 1.0 × 10^5^ cells/mL in a vial. Briefly, 1.0 × 10^4^ of mouse pre-osteoblasts were suspended in 100 μL of culture medium (EMEM, 10% FBS, 1% antibiotic solution) and seeded into each well of a 96-well cell grade Brand microplate (adherent wells) and cultured to a semi-confluent monolayer at 37 °C, 95% humidity, and 5% CO_2_ in an incubator for 24 h. The cement pastes were immersed in culture medium (0.2 g/mL of culture medium) (according to ISO 10993-12:2012) [22] for 24 h at 37 °C. Subsequently, the culture medium in the wells was replaced with 100 μL of 100% extract. All experiments were carried out in triplicate, and cells in wells with extract-free complete culture medium were considered as the negative control (NC). After 24 h of culture, the extracts were replaced with fresh culture medium, and in vitro cytotoxicity was evaluated (ISO 10993-5:2009) [23] using the MTS proliferation test assay (Cell titer 96 aqueous one solution cell proliferation assay, Promega Madison, WI, USA) and a UV–VIS spectrophotometer (Shimadzu, Kyoto, Japan) at 490 nm.

The determination of the long-term cytotoxicity of cement–honey pastes was carried out after 7 and 14 days of rat MSCs cultivation. The rat MSCs were isolated from femur and tibia bone marrow. The MSC phenotype was confirmed using flow cytometry after the staining of cell-specific markers (CD45/CD29/CD90.1, eBioscience, San Diego, CA, USA) and differentiated cells for the confirmation of their multi-differentiation capacities (StemPro Osteogenesis, Chondrogenesis and Adipogenesis Differentiation Kit (GIBCO), Waltham, MA, USA) [24].

For the long-term cytotoxicity testing of extracts (up to 14 days), 0.1 g cement/mL medium was used. The cement pastes were soaked in complete osteogenic differentiation culture medium (α-modification Eagle’s Minimum essential medium (αMEM), 10% FBS, osteogenic supplements: 50 μg/mL of L-ascorbic acid, 50 nM dexamethasone, 10 mM β-glycerophosphate, and 1% antibiotic solution, all of which were acquired from Sigma-Aldrich, Saint Louis, MO, USA) in an incubator at 37 °C for 24 h [22]. The rat MSCs at passage 3 were resuspended in culture medium after harvesting via enzymatic digestion, and cell density was adjusted to 5.0 × 10^4^ cells/mL in a vial. Briefly, 2.0 × 10^4^ of rat MSCs were suspended in 400 μL of EMEM + 10% FBS and 1% antibiotic solution and seeded into each well of a 48-well plate and cultured to a semi-confluent monolayer at 37 °C, 95% humidity, and 5% CO_2_ in an incubator for 24 h. Subsequently, the culture medium in the wells was replaced with 400 μL of prepared extract (0.1 g/mL). All experiments were carried out in triplicate, and cells in the wells with extract-free complete culture medium were considered to be the negative control (NC). Extracts were refreshed every two days.

The ALP activity of osteoblastic differentiated rat MSCs (after 7 and 14 days cultivation) was determined from cell lysates after lysis with solution containing 0.1% Triton X-100. The cell lysates were frozen at −20 °C after thawing and centrifuged at 10,000 RPM for 10 min. Aliquots of the cell supernatant were added to p-nitrophenyl phosphate in dietanolamine buffer, and after incubation for 60 min at 37 °C, the amount of p-nitrophenol produced by ALP enzyme catalysis was determined from the calibration curve of p-nitrophenol at 405 nm using a UV-VIS spectrophotometer. The ALP activities were expressed in micromoles of the p-nitrophenol produced per 1 min per microgram of proteins. The protein content in the lysates was determined using Bradford’s method with Coomasie blue G250 as the complexing agent.

The Alizarin red staining of calcium deposits produced by differentiated osteoblasts was carried out after the extracts had been subjected to rat MSC cultivation for 7 and 14 days. Cells were washed with PBS, fixed in ethanol, and washed with distilled water. Deposits were stained with Alizarin red S staining solution for 30 min. After the removal of the staining solution and washing three times with distilled water, the cells were observed under a light microscope (Leica DM IL LED, Heerbrugg, Switzerland).

#### 2.3.1. Analysis of Osteogenic Gene Expression in Differentiated Rat MSCs

Gene expression was analyzed according to the method outlined in [6]. For the extraction of total RNA, 1.0 × 10^6^ cells were used. Total RNA from each cell culture was extracted using a RNeasy Mini Kit (Qiagen, Germantown, MD, USA) following the manufacturer’s instructions. Contaminating genomic DNA was digested using an RNase-free DNase set (Qiagen, Germantown, MD, USA). The RNA quality and yields were analyzed using a NanoDrop spectrophotometer (Thermo Scientific, Waltham, MA, USA). Complementary DNA (cDNA) synthesis was performed using the protocol for the RT2First Strand Kit (Qiagen, Germantown, MD, USA), where 1 μg of total RNA was used (after the genomic DNA elimination step) to prepare 20 μL of cDNA, which was used for real-time PCR experiments (RT PCR). The quantification of genes of interest in the cDNA samples was performed using primers for the following genes: B-actin rat, type I collagen rat, osteocalcin rat, osteopontin rat, and osteonectin rat (Table 1). A 25-μL reaction mixture consisting of triplicate samples of cDNA, specific primer mix, and RT2 SYBR Green qPCR mastermix (Qiagen, Germantown, MD, USA) was set up in each well of a 96-well reaction plate (Roche, Basel, Switzerland). cDNA for β actin was used as the endogenous control for calculating fold differences in the RNA levels of cells treated vs. not treated with cement extracts according to the 2^−∆∆CT^ method. The plate was sealed using an optical adhesive cover (Roche, Switzerland) and placed in a LightCycler 480 II RT PCR system machine (Roche, Switzerland). RT PCR was performed under the following conditions: initial incubation at 95 °C for 10 min, followed by amplification in 45 cycles at 95 °C for 15 s followed by 60 °C for 1 min. Amplification specificity was checked via the generation of a melting curve.

#### 2.3.2. Antibacterial Activity of Cements and Honey Samples

The antibacterial activity of honey cement samples was investigated by using two bacterial strains: Gram-negative *Escherichia coli* ATCC 11303 and Gram-positive *Staphylococcus aureus* CCM 5776 (Czech Collection of Microorganisms, Brno, Czech Republic). The *E. coli* ATCC 11303 and *S. aureus* CCM 5776 strains were inoculated onto the surface of nutrient agar (OXOID, Cheshire, UK) and cultured at 37 °C for 24 h. From the 24 h culture, the bacterial colonies were suspended in brain–heart infusion broth (BHI), and the turbidity was adjusted to 0.5 degrees (1.0 × 10^8^ CFU.mL^−1^) according to the McFarland turbidity standard using a densitometer (BIOSAN, Riga, Latvia).

The tested samples were used in the following forms: 1 mL of undiluted medical honey, 1 g of TTCPM cement paste, 1 g of V10 or M10 paste. The pastes were prepared from sterile powders and liquids under aseptic conditions. A total of 3 mL of BHI broth bacterial suspension was added to each freshly prepared sample and mixed. BHI broth (3 mL) with *S. aureus* and *E. coli* was considered as the negative control (NC); BHI broth (3 mL) without bacterial strains was considered as blank. The final volume of bacterial suspension in all samples was 3 mL. The bacteria samples were cultivated in an incubator at 37 °C for 24 h. After rigorous mixing, 200 µL suspension was removed from each bacteria–cement suspension and transferred into a 48-well plate, and MTS reagent (Cell Titer Aqueous One Solution Cell Proliferation Assay, Promega, Madison, WI, USA) was added according to the manufacturer’s instructions. After 30 min incubation at 37 °C, the absorbance of formazan metabolized by active bacteria from tetrazolium was measured using a UV-VIS spectrophotometer at 490 nm (Shimadzu, Kyoto, Japan). The relative inhibition of bacterial activity was calculated as the difference between the NC formazan absorbance (set to 1 or 100%) and the ratio of the formazan absorbance reduced by the blank value in the tested samples to the NC formazan absorbance.

## 3. Results

### 3.1. XRD and FTIR Analysis of Cements

**Figure 1:**
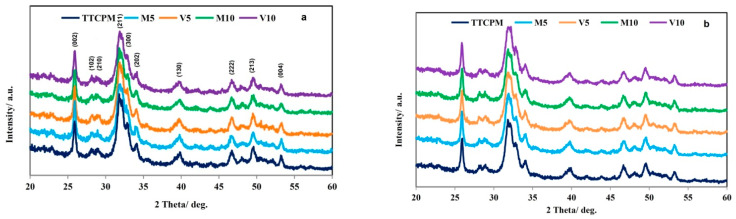

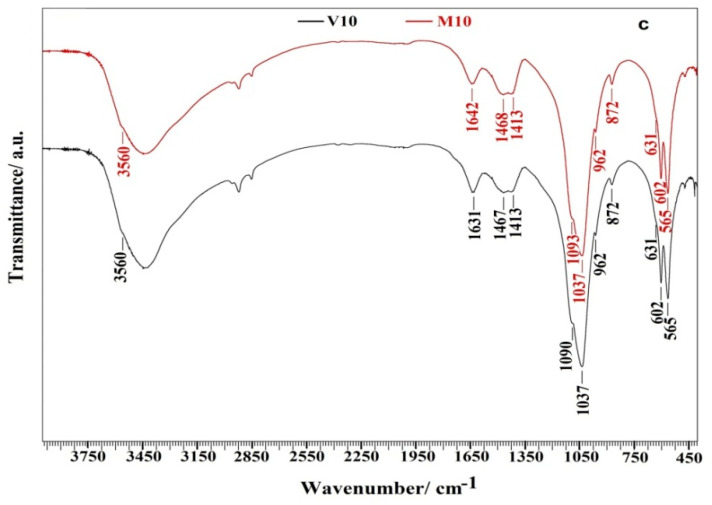
XRD patterns of TTCPM and honey cements after 1 (**a**) and 7 (**b**) days of soaking at 37 °C in SBF (identified full transformation to nanocrystalline calcium-deficient hydroxyapatite (assigned lines of HAP (PDF4 01-071-5048)) after 24 h without any secondary phases or origin calcium phosphates regardless of honey addition) and FTIR spectra (**c**) of M10 and V10 cements after 7 days of soaking at 37 °C in SBF (clearly showed the formation of carbonated HAP).

The XRD patterns of the cements after hardening for 1 and 7 days in SBF at 37 °C are shown in Figure 1a,b. The nanocrystalline hydroxyapatite (HAP) (PDF4 01-071-5048) was identified in all patterns, and no traces of other secondary phases that arise from starting calcium phosphate components in origin powder cement mixture or their transformation after immersion in SBF were found. The chemical analysis of the final nanohydroxyapatite phase by ICP demonstrated the formation of calcium-deficient hydroxyapatite with a Ca/P molar ratio equal 1.64 ± 0.02. The average crystallite size of nanohydroxyapatite in hardened cement was calculated from the line representing the reflection of (002) the HAP plane using Scherrer´s equation. The results showed that crystallite size was not dependent on the addition of honey to the cements, and the average crystallite sizes were around 29 and 33 nm for the hardened cements after 1 and 7 days, respectively.

FTIR analysis of the M10 and V10 cements after 7 days of soaking in SBF at 37 °C verified the results from XRD analysis (Figure 1c). The insufficiently distinguished peaks in the spectra, characteristic of the stretching and liberational modes of the OH group at 3560 and 630 cm^−1^, respectively, clearly revealed the nanocrystalline nature of HAP. Moreover, the presence of HAP confirmed the peaks at 962 cm^−1^ (ν_1_ stretching symmetric vibrations), 602 and 564 cm^−1^ (O–P–O bending vibrations from triplicate degenerate bending ν_4_ mode), and 1090 and 1037 cm^−1^ (triple-degenerated antisymmetric stretching ν_3_ mode) from PO_4_^3-^ of hydroxyapatite [30]. In addition, bands at 1471, 1423, and 872 cm^−1^ representing asymmetric stretching ν_3_ and ν_2_ (out-of-plane bending) vibrational modes of the carbonate group in B-type carbonated hydroxyapatite were found in all spectra [30].

### 3.2. Microstructures of Cements and Morphology of HAP Particles in Cements, Setting Time, and Compressive Strength of Cements

**Figure 2:**
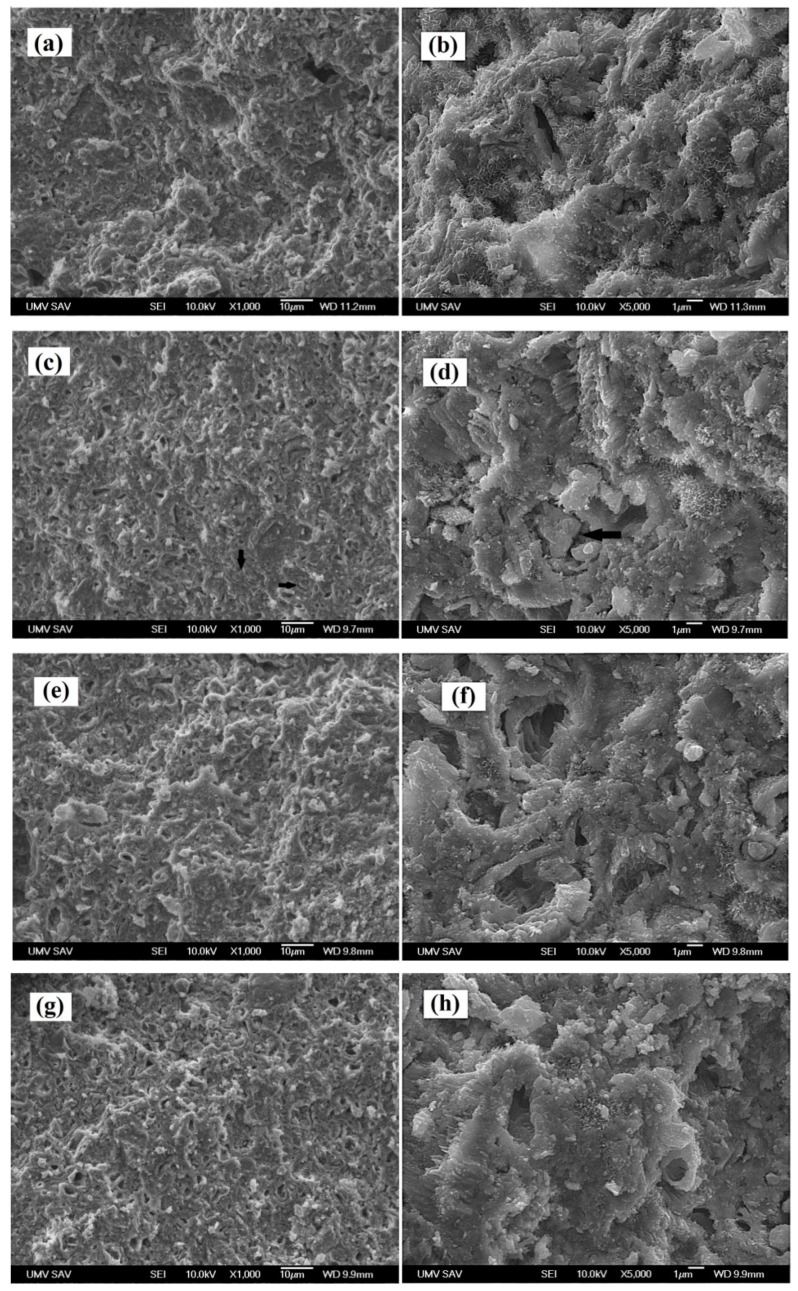

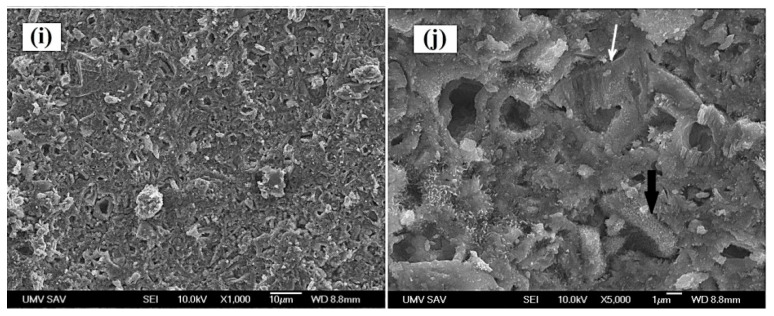
Microstructures of cements after hardening in SBF at 37 °C for 7 days ((**a**,**b**)—TTCPM; (**c**,**d**)—M5; (**e**,**f**)—V5; (**g**,**h**)—M10; (**i**,**j**)—V10). White arrow—columnar growth of HAP particles; black arrows—agglomerates of HAP nanoparticles weakly bound to the cement matrix due to their separation by the gap zone.

The microstructures of the cements after hardening in SBF at 37 °C for 7 days are shown in Figure 2. A high fraction of fine 1–2 µm pores and several larger irregularly shaped macropores up to 10 µm in size were visible in the microstructure of TTCPM cement (Figure 2a). HAP particles were joined to coarser and more compact globular agglomerates (up to 10 µm size) that were pulled out of the matrix after fracture. In addition, the HAP particles in the agglomerates had a rod-like morphology with a length of up to 400 nm, and a small fraction of very fine particles with a spherical morphology and submicron size were visible in Figure 2b. In the case of the honey cement samples, after pulling out the agglomerates from the cement matrix, a greater amount of irregularly shaped micropores (size 2–3 µm) were identified in the microstructures (Figure 2c,e); however, upon more detailed analysis (Figure 2d,f), the images showed a columnar growth of long needle-like HAP particles (up to 1 µm in length). The columnary arranged particles formed walls around the original calcium phosphate (probably TTCP) particles which were transformed into nanocrystalline HAP. Moreover, in detailed micrographs of the M5 or V10 microstructures, the agglomerates of newly formed HAP particles were often even separated from the surrounding matrix by a cement gap zone with a weak connection at the agglomerate/matrix boundary (Figure 2j).

**Figure 3:**
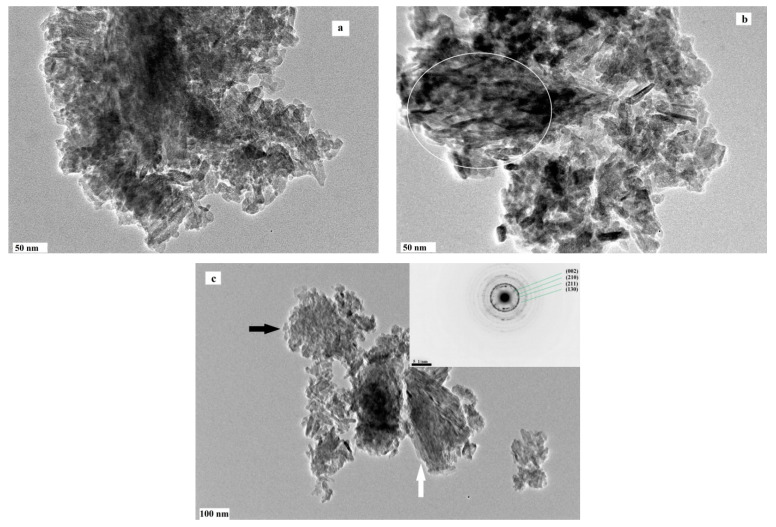
Morphology of HAP particles in cements after 7 days of soaking at 37 °C in SBF ((**a**)—M5; (**b**,**c**)—M10; selected area electron diffraction in detail with corresponding HAP lines; white arrow—part of pore wall with columnarly grown HAP particles; black arrow—agglomerate of very fine 10–20 nm globular HAP particles; white circle—agglomerate of needle-like HAP particles).

The morphology of the HAP particles in the M5 and M10 cements after 7 days of soaking in SBF was characterized via TEM (Figure 3). The needle-like morphology of HAP prevailed in cements regardless of the kind or concentration of honey in the cement matrix. Additionally, a lower number of very fine spherical HAP particles (5–10 nm in size) was observed in the cements. The remains of the walls with columnary arranged needle-like HAP particles originally found in the microstructures around the pores can be found in Figure 3c (particle agglomerate of ~300 nm size).

The apparent density of the hardened cements was independent following the addition of honey to the cement and was close to 52 ± 2% of theoretical HAP density. The CS of the TTCPM cement was 42 ±2 MPa and decreased with 5% (*w*/*v*) honey addition down to 35 ± 5 MPa and 26 ± 4 MPa for V10 and M10 cements, respectively. The final setting time of the TTCPM and honey cements was 4 ± 1 min and independent of the type of honey addition used.

### 3.3. Changes in pH and Release of Ca and Phosphate Ions during Soaking in Aqueous Solution

**Figure 4:**
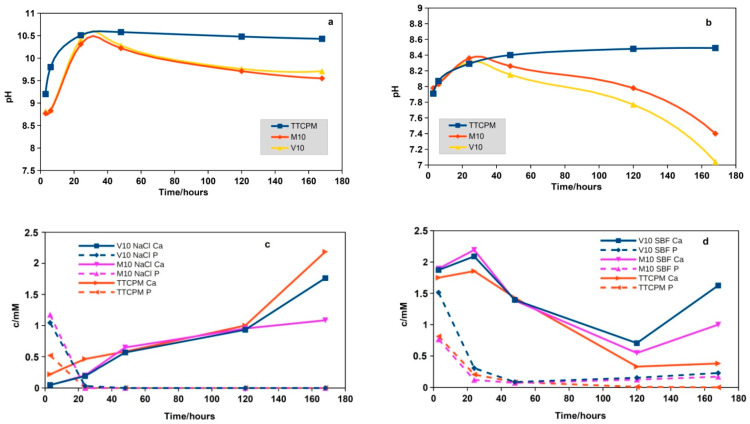
Changes in pH and calcium phosphate ion concentrations of saline (**a**,**c**) and SBF (**b**,**d**) solutions during the soaking of cements at 37 °C (reduction in pH during cement setting after hydrolysis of TTCP and interaction with monetite to final HAP product (with corresponding changes in calcium and phosphate concentrations in solutions, probably due to the formation of surface complexes and the ionization of polyphenols in acidic conditions, as well as the oxidation of sugars to organic acids).

Adding honey to the liquid components insignificantly influenced their pH (close to 4.4). Measuring the pH changes during cement soaking in saline can help identify the physicochemical processes that are not affected by the buffering capacity of any additives that are added to the SBF. From the comparison of the individual curves in Figure 4a,b, it is clear that the dependencies of pH on the soaking time of TTCPM and honey cements were different. A rapid increase in pH was observed for the TTCPM and honey cements; pH rose up to 10.5 and 8.4 during the first 24 h of soaking in NaCl and SBF, respectively, though this subsequently stabilized for TTCPM (to 10.5 or 8.4 in NaCl and SBF), and a gradual decrease for the honey cements (to 9.5 and 7 in NaCl and SBF) can be identified using the curves.

The changes in pH were accompanied by a rapid reduction in phosphate concentration in both media (NaCl or SBF solutions), regardless of they the type of honey that was added to the cement (Figure 4c,d). On the other hand, the calcium concentrations in NaCl solution rose with soaking time, contrary to that in SBF solution, where initially a small increase to about 2.2 mM was found after 24 h, followed by a decrease to 0.8 and 0.5 mM after 120 h from immersion for the TTCPM and honey cements, respectively. During prolonged soaking times, the rise in calcium concentration was verified in solutions with honey cements, whereas the Ca concentration in the TTCPM SBF solution was almost constant (~0.3 mM).

### 3.4. Analysis of Proteins, Release of Polyphenols and Sugars from Honey Cements, and Antioxidant Capacity of Cement Extracts

**Figure 5:**
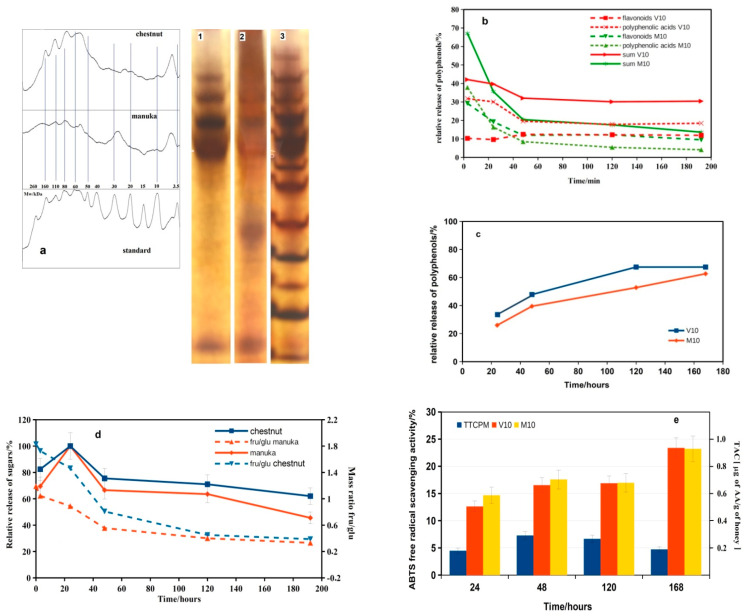
Protein profiles of honeys (determined via SDS PAGE electrophoresis). (**a**) lane 1: chestnut, lane 2: manuka, lane 3: protein standard; relative release of polyphenols and flavonoids from honey cements to physiological (**b**) and SBF (**c**) solutions demonstrate the strong adsorption of polyphenols on the surface of HAP after ionization at basic conditions followed their gradual desorption with increasing in pH; the relative release of sugars and fru/glu mass ratio during soaking for the honey cements in the physiological solution (**d**) indicated the significant adsorption of both sugars after the transformation of cements and a decrease in fru content in relation to glu with soaking time (probably the result of oxidation and degradation due to low molecular organic acids on active HAP sites); changes in the ABTS free radical scavenging activities and TAC of SBF the cement extracts with soaking time (**e**) verified the rapid increase in FRSA in the honey composites, as well as its stability, regardless of soaking time.

The total proteins contents in the manuka-based and chestnut honey were 0.13 ± 0.02 wt% and 0.07 ± 0.015 wt%, respectively. The protein profiles of the honeys, which were determined via SDS PAGE electrophoresis, are shown in Figure 5a. Our analysis revealed the presence of proteins with M_w_ equal to 6, 10, 25, 55, 78, 110, and 160 kDa in both honeys, but the manuka-based honey contained a high number of proteins with M_w_ of about 26 kDa. The identified proteins represent the major royal jelly proteins with M_w_ ~ 48–70 kDa and proteins originated from nectars or pollen between M_w_ 20–30 kDa, and proteins with M_w_ equal 5 and 10 kDa can be assigned to apisimin and defensin-1 [31,32]. The total polyphenol contents of the honeys, which were determined colorimetrically, were 1.45 and 0.99 mg/g for the manuka and chestnut honeys, respectively. Moreover, the flavonoid contents of the two honeys were 0.87 and 0.39 mg/g of honey. The amounts of selected polyphenols and flavonoids (determined using HPLC method) in both honeys are shown in Table 2.

A decrease in the amount of polyphenols and flavonoids released from the honey cements was revealed during the first 48 h (from immersion to physiological solution) (Figure 5b). After 3 h of soaking, about 70% and 42% of the total amount of polyphenols were released from the M10 and V10 cements, respectively, to the solution. On the other hand, only a very slow reduction in the amount of released polyphenols (2–5%) was identified in both cements, and 15 and 32% of the polyphenols were released from the V10 and M10 cements after 192 h. Regarding the cement extracts in SBF (Figure 5c), there was almost no difference in the amount of polyphenols released from the V10 and M10 cements (around 30% of the total polyphenols were released to SBF), and this was confirmed after 24 h of immersion. In addition, a gradual increase in the amount of released polyphenols with soaking time was observed for both cements, and ~60% of the total polyphenols were released into the SBF after 168 h of soaking.

The individual sugar contents of the honeys are shown in Table 2. Regarding the individual sugars (fructose (fru) and glucose (glu)), 100% of the total sugar content in the cements was released into the physiological solution after 24 h of immersion (Figure 5d). The released amount of sugars from the M10 and V10 cements rapidly reduced to about 65 and 75%, respectively, with a subsequent gradual decrease down to 45 and 63% during prolonged soaking for 192 h. In addition, the rapid decline in fru/glu mass ratio in the extracts was measured during the first 48 h of soaking; the V10 extract went from having a fructose content value that was almost double that of glu (fru/glu = 1.8) in chestnut honey to a fructose content of 0.8, and this gradually declined further to ~0.4. Moreover, almost the same dependence of fru/glu mass ratio was found in manuka honey, with a decrease in ratio to about 0.4 in the M10 extract, followed by a further gradual reduction to 0.3 after 192 h of soaking.

The free radical scavenging activities (FRSA) of the chestnut and manuka honeys characterized 38 ± 2 and 41 ± 2% of the radical inhibition determined via the DPPH method and 33 ± 2 and 72 ± 3% determined via the ABTS method, respectively. In addition, the TAC values (analyzed using the ABTS method) were 1.30 ± 0.05 and 2.76 ± 0.05 mg of AA/g for the chestnut and manuka honeys. The dependence of the FRSA (ABTS method) of the SBF cement extracts on soaking time (Figure 5e) revealed a gradual increase in value to about 17% after 48 h of soaking, and the FRSA was confirmed to be almost stable up to 120 h, with a subsequent increase to about 23% occurring after 168 h of immersion. The TAC of the SBF honey extracts achieved approx.. 0.9 mg of AA/g of honey after 168 h soaking.

### 3.5. In Vitro Cytotoxicity Testing of Cements and Cement Extracts, Live/Dead Staining, and Gene Expression of Cell in Cement Extracts

**Figure 6:**
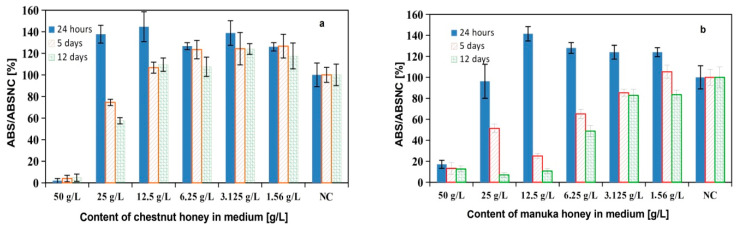

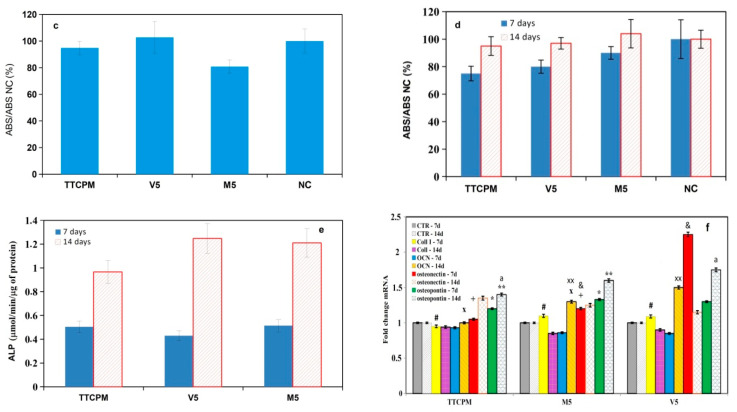
Cytotoxicity of medium at different concentrations of honey (**a**,**b**); cytotoxicity of TTCPM, V5, and M5 cement extracts after 24 h of cultivation (**c**) and long-term cultivation of cells for 7 and 14 days in the cement extracts (**d**); ALP activities of MSC´s cultured in cement extracts for 7 and 14 days (**e**); RT PCR analysis of osteogenic gene expression in cells cultured in cement extracts for 7 and 14 days. (**f**) Statistically significant differences between the expression of osteogenic genes in the cells cultured in the honey cement extracts and that in the cells cultured in the TTCPM extract and the upregulation of the osteocalcin (OCN), osteopontin (OP) (#, *, **, X, XX, a: *p* < 0.001), and the osteonectin (ON) genes (&, +: *p* < 0.0001) for the V5 extract in the cells that were cultured in the honey cement extracts for 14 days and the cells that were cultured in the honey cement extracts for 7 days.

Before starting the experiments with the honeys, the maximum concentration of honey in the liquid component of the cements was optimized based on the measurement of the medium cytotoxicity at different concentrations of honey in the medium (Figure 6a,b). Because the lowest noncytotoxic concentrations of honey were very different for the chestnut and manuka honeys, the lower concentration equal to 3.125 g of honey/L of solution was selected as the limit concentration of possible cytotoxicity for the cements for in vitro testing after immersion in the solution. This fact corresponds to the applied max. 10% (*w*/*v*) honey content in the liquid component of the cement used to analyze the material properties of the cements; however, in vitro testing was performed with cements containing 5% (*w*/*v*) honey in the liquid component so as to not use limit concentrations for cytotoxicity.

No cytotoxicity was found in the TTCPM, V5, and M5 cement extracts after 24 h of cultivation (measured using MTS test according to ISO10993-5) [23] (Figure 6c) (<70% viability of cells in relation to NC was selected as the cytotoxicity criterion for the tested samples). Regarding the long-term proliferation of rat MSCs in the extracts, a strong rise in the proliferation of cells with culture time in cement extracts was verified, which revealed the non-cytotoxic character of extracts even after a long period of cultivation (Figure 6d). The ALP activities of the MSCs cultured in the cement extracts rose with culture time and were approx. double and almost triple for the cells cultured in the TTCPM, V5, and M5 extracts, respectively (Figure 6e). The increase in the osteogenic potential of the MSCs during cultivation in both honey cement extracts indicated the upregulation of osteogenic genes like osteocalcin (OCN) and osteopontin (OP) after 14 days and the upregulation of osteonectin (ON) genes after 7 days of cultivation in honey cement extracts (Figure 6f) compared with the cells cultured in the TTCPM extract.

**Figure 7:**
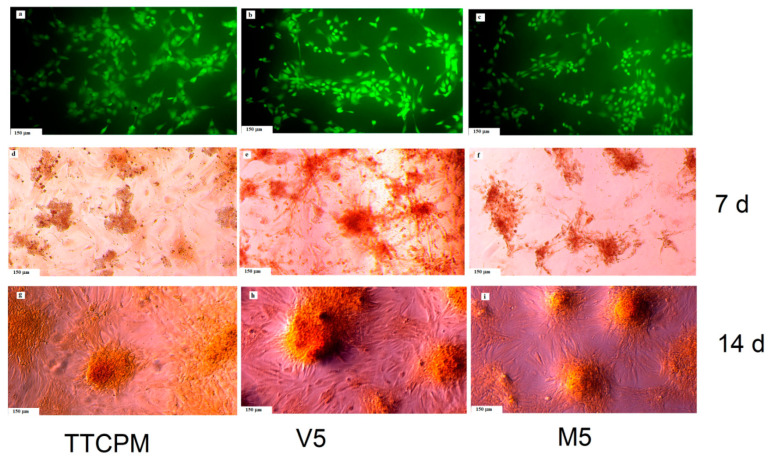
Live/dead staining of MC3T3E1 cells on surface of cement samples after 48 h of cultivation in cement extracts (**a**–**c**) indicated the good adherence of the cells to cement surfaces with no visible dead cells; calcium deposits produced by cells cultured in cement extracts for 7 and 14 days stained by alizarin red (**d**–**i**) (with a higher production in the case of composite cement extracts).

The images derived from our live/dead staining (Figure 7a–c) clearly demonstrate the good adherence of MC3T3E1 cells on the surface of the cement samples after 48 h of cultivation, as no dead cells were found. The density of the cells and their distribution on the surfaces were almost identical for the cements; the cells were well spread and partially connected by filopodia, which confirmed the non-cytotoxic nature of the sample surfaces, regardless of the cement type. Alizarin red staining confirmed the ability of differentiated rat MSCs to produce calcium deposits (Figure 7d–i).

### 3.6. Antibacterial Activity of Cements

**Figure 8:**
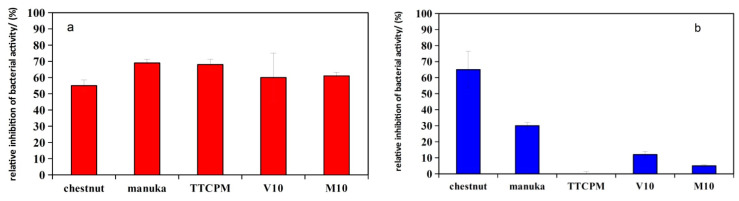
The relative inhibition of bacterial activity of the cement extracts for *Escherichia coli* (**a**) and *Staphylococcus aureus* (**b**) in the cement samples revealed that the RIA values of the cement extracts were close regardless of the type of honey added to the cement for *E. Coli*, which clearly confirms the key effect of cement base on RIA. The RIA of the TTCPM extract for *S.Aureus* was almost zero in contrast to the relatively high RIA of the liquid components containing the honey and composite extracts, which released <50% of the total amount of polyphenols in the cements.

The antibacterial activity of the TTCP/honey paste was examined by using two bacterial strains: *Escherichia coli* as a Gram-negative strain and *Staphylococcus aureus* as a Gram-positive strain. The *E. coli* was more sensitive to the cement extracts and hardening liquid with honeys. In the case of *E. coli*, the relative inhibition of bacterial activity (RIA) of all of the tested samples represents an interval within a range of 55–70% in relation to NC, and this contrasted with that of *S. Aureus*, where the RIA´s of the V10 and M10 cement extracts were 12 and 5% (statistically significant difference, *p* < 0.05), respectively, while the TTCPM extract had almost zero RIA. The liquid components of the chestnut and manuka honeys had RIA´s of 55 and 70% for *E. Coli* (statistically significant difference, *p* < 0.05) and 65 and 30% for *S. Aureus,* respectively (Figure 8a,b).

## 4. Discussion

To the best of our knowledge, there is practically no information in the literature about the influence of honey on the material characteristics or properties of CPC´s as well as on the behavior of osteoblastic cells in vitro after adding honey to CPC´s. Honeys contain a high concentration of sugars, especially fructose and glucose, as this was measured in the manuka and chestnut honeys (82 and 87 wt% of sugars, respectively), while the fraction of other active ingredients, such as proteins, polyphenolic acids, or flavonoids, did not exceed tenths of wt%. It is clear that the microstructure of honey cements can be affected by the above-mentioned compounds. The monosaccharides effectively reduced the growth of HAP particles even in a very low concentration in the supersaturated solutions of calcium phosphates during precipitation due to both a strong affinity to HAP and the negative shift in the zeta potential of particles [33]. The formation of needle-like HAP particles was demonstrated after adding glucose to the reaction mixture under alkaline conditions at 70 °C, and results showed that the higher temperature of precipitation supported the elongation and growth of HAP crystals along the c-axis [34]. On the other hand, the stable complexes between the Ca^2+^ or phosphate ions and glucose were verified in solutions during HAP precipitation under neutral conditions, which caused the refining of HAP particles. Refinement and a decrease in crystallite size in the c-axis were even found after adding glucose to freshly precipitated HAP particles, where adsorbed glucose strongly interacts with OH groups in HAP and the formation of both the calcium hydroxide and calcium carbonate was indicated [35]. Flavonoids reduced the crystallite size of HAP particles at a low concentration in reaction solution during the neutralization reaction without preferential growth, but no changes in morphology were confirmed after their adsorption on the surface of particles [36]. In the case of using gallic acid as an example of polyphenolic acid, a strong effect on crystallite size was revealed with the formation of Ca/gallic acid complexes, and an increase in the Ca/P ratio due to the replacement of phosphate functional groups in the crystal lattice was found [37]. Similarly, the effective inhibition of HAP precipitation as well as HAP crystal growth in the presence of polyphenols with a larger molecular weight (tannic acid, humic acid) was verified, and we believe that this was the result of energetically favorable adsorption on the surface of HAP nuclei [38]. Note that the preferential oriented HAP along the c-axis was synthesized from αTCP via a hydrothermal process using flavonoid—quercetin as a template in relative high amounts [39]. The crystallite size of the HAP particles and the setting process were not affected by the presence of honey during the transformation process of TTCPM cement, but the morphology and recrystallization of the HAP particles were influenced by the addition of honey. The columnary growth of long HAP particles with a needle-like morphology was found in honey cements, which could be related to the presence of monosaccharides in the cement matrix. The rapid release of monosaccharides (up to 100% of the total content in cement) was measured during the first 24 h of immersing the honey cements in NaCl solution, with a subsequent decrease to 70–80% total sugar content being observed in the cements after 48 h of soaking. Thus, approx. 20–30% of the sugars were adsorbed in hardened and transformed cement on surfaces of HAP particles when the origin calcium phosphate phases were transformed to nanohydroxyapatite. The low adsorption of glucose and fructose proceed on the surfaces of active powdered calcium phosphate particles due to fast changes in the chemical character and texture of the surface of particles during the hydrolysis of TTCP, and the formation of calcium hydroxide is accompanied by a rise in pH. Moreover, the nucleation of HAP particles followed the dissolution of the monetite nanoparticles (the change in phosphate concentration in solution) and mutual interactions. These processes clearly documented the dependences of the changes in the concentration of calcium and phosphate ions during the first 24 h of immersing the cements into solutions, and a rapid rise in phosphate concentration and slow increase in calcium concentration were identified after 3 h. It should be noted that the catalytic effect of HAP on the transformation of monosaccharides can probably be predicted by measuring the decrease in fru/glu mass ratio with soaking time. It is known that reducing sugars creates α-dicarbonyl compounds and, consequently, calcium complexes [40], and glucose is isomerized to fructose, which is more easily decomposed to lower organic acids and reduces the pH of the media [41]; this has been observed in honey cement extracts. Despite the possible formation of Ca-monosaccharide complexes in a local medium with a sufficient concentration of phosphate ions due to the increased solubility of monetite, these complexes are not very stable and decompose with the formation of poorly soluble hydroxyapatite, which is also supported by the fact that the addition of honey had a negligible effect on the setting time. A similar dependence was revealed in the case of polyphenols, especially polyphenolic acids, which were gradually dissociated with the increase in medium basicity and adsorbed on the surface of HAP particles. These facts were supported by a gradual decline in pH with prolonged soaking in both aqueous media due to the interaction of free hydrogen ions with hydroxide ions. On the other hand, the amount of polyphenols released to SBF increased with soaking time due to recrystallization of HAP, which initially caused the loss of active surface sites on particles and subsequently caused a partial decrease in ionization with the increasing solubility of the surface complexes and HAP nanoparticles in a more acidic SBF. It is clear that the formation of surface complexes decelerates the diffusion of ions on longer distances, and the local supersaturation related to the HAP solubility constant is achieved relatively quickly on the adjacent surfaces of particles surrounded by a more acidic environment, and recrystallization to columnar form is also controlled with the participation of adsorbed chemical species. The higher concentration of calcium ions found in the SBF solutions compared with the TTCPM cement after soaking the honey cements for 168 h verifies the above conclusion. The local increase in pH could be the reason behind the presence of the gap zone that separates the agglomerates of fine globular nanoparticles from the columnar growing needle-like HAP particles as a result of the higher solubility of the globular particles. In addition, the weaker bond between the HAP agglomerates and the cement matrix, as well as the adsorption of various organic substances on the surfaces of the HAP particles, probably reduced the cohesion of nanoparticles and the overall strength of cement matrix, which documented the decrease in the compressive strength of honey cements. In the case of soaking in NaCl solution, the Ca and phosphate concentrations were very close after the full transformation of the starting calcium phosphates in the cements and slowly rose with soaking time up to 120 h, but the solubility of HAP particles in almost steady-state condition after 168 h soaking descended in an order (TTCPM > V10 > M10) that corresponds with the amount of polyphenols adsorbed on honey cements and the resultant density of the surface complexes. It should be noted that, in addition to COOH groups, phenolic OH groups also dissociate at pH > 9 [38], which strengthens the surface interaction with calcium ions. On other side, a part of the phenolic compounds is incorporated into the HAP lattice, and it is difficult to release them into the solution [36].

The ABTS FRSA of the honey extracts achieved about 23% of the antioxidant inhibitory activity, and TAC was close to 0.9 mg AA/g of honey, which was about 30% and 70% of manuka and chestnut honey TAC, respectively. The changes in FRSA correlated quite well with the amount of polyphenols released (around 50% of total content) into the SBF solution (the linear regression coefficient equalled 0.95 and 0.90 for the V10 and M10 cements, respectively). However, the FRSA of the TTCPM extract increased significantly (more than three-fold) after the addition of honey, indicating the direct effect of honey components on the antioxidant properties of the cements. Both honeys had relatively high TAC values (1.3 and 2.7 mg of AA/g of honey) compared with Czech or Croatian monofloral honeys (0.4 mg of AA/g of honey), but honeys from other sources (heterofloral, raspberry, honeydew, etc.) had TAC values up to 0.9 mg of AA/g of honey. On the other hand, the total phenolic content was much lower in these honeys (especially in monofloral), excluding honeydew honey, which had 1.2 mg of GA/g of honey [42,43]. In the case of Algerian floral honeys with phenolic contents (0.9–1.5 mg of GA/g of honey) comparable to that used in the manuka and chestnut honeys of this study, similar ABTS antioxidant inhibitory activities of ~20–50% were found [19]. It is clear from the above that, despite the lower antioxidant inhibitory activity of the honey in the cement extracts compared to the pure honey solutions, ABTS FRSA was close to the common monofloral or heterofloral honeys.

The main difference between Gram-negative and Gram-positive bacteria is in the structure of the cell wall, where a thick layer of peptidoglycane characterizes the Gram-positive bacteria, and thin peptidoglycane surrounded by a lipopolysacharide layer in the outer membrane represents the Gram-negative bacteria. These differences in the cell well structure can affect the sensitivity of bacteria to antibacterial substances, including honeys. It has been shown that the addition of heavy metal ions like Ag, Zn, and Bi to CPC [7,44,45,46] and the formation of specific nano/micro-topographies on final cement surfaces (pillars and rod- or needle-like particles) can effectively enhance the antimicrobial activity of CPC´s [47]. Moreover, the antibacterial activity of the CPC´s was revealed, and calcium hydroxide is one of the products of the setting process in the mixture of dihydrogen phosphate monohydrate and calcium oxide with sodium phosphate buffer in a liquid phase, where clear and reproducible bacterial growth inhibition was observed against all the microorganisms tested for the cement samples with Ca/P ≥ 2 [8]. Similarly, inhibitory activity was observed for nanocrystalline tetracalcium phosphate cement, with > triple the inhibition zones for *S. Epidermidis* and *S. Salivarius* being detected around the cement samples compared to crystalline Ca(OH)_2_ using agar diffusion tests due to the formation of amorphous Ca(OH)_2_ in the cements [48]. Moreover, the inhibition efficiencies of the micro-layered calcium hydroxide nanoparticles were 63% and 50% for *S. aureus* and *P. Putida*, respectively, and reactive oxygen species-mediated antibacterial proficiency was also demonstrated [49]. The alkaline ions containing CPC´s showed a significantly higher antimicrobial efficiency (almost tenfold) than the commercial calcium hydroxide cement, which was a result of the enhanced solubility of alkali metal phosphates and the subsequent formation of corresponding hydroxides with an increase in pH [50]. Regarding the TTCMH cement, the pH of the SBF solution and amount of calcium hydroxide increased during the first 24 h of cement immersion; however, following the addition of honey to the cement, a gradual reduction was recorded (probably due to the consumption of hydroxyl ions in the solution). The RIA´s for *E. coli* were close to 50–60% in all tested samples, and from our comparison, honey addition seemingly had a small effect on RIA. Therefore, the antibacterial activity of cements for *E. coli* can be related to the release of Ca(OH)_2_ during cement transformation. Absolutely opposite results were revealed for *S. aureus*, where, despite the fact that the antimicrobial activity for the TTCPM cement was very low, the V10 and M10 cements had RIA values that statistically significantly higher than 0 (*p* < 0.05) but much lower than the RIA values of the chestnut or manuka honey solutions (statistically significant difference, *p* < 0.001). These results indicate the direct effect of the addition of honey on the antimicrobial properties of the cements with respect to *S. Aureus*, which may be a consequence of the amount of polyphenols released from the cements and the concentration of methylglyoxal or H_2_O_2_ in the extracts. Manuka honey has been shown to eradicate methicillin-resistant *S. aureus* and have a synergistic effect with antibiotics to improve antimicrobial properties [51]. In addition, it had a higher antimicrobial activity against Gram-positive bacteria compared to Gram-negative bacteria (*E. coli*) [52]. Regarding bioactive materials, the relative viability of bacteria in media with bioglass/methylcellulose foams after the addition of 2.5 wt% of manuka honey significantly decreased *S. aureus*, and an even a stronger effect was observed on *E. Coli* [53]. Moreover, the 45S5 bioactive glass-based scaffolds coated with corn protein zein and Manuka honey showed antibacterial activity against *S. aureus*, with about a 70% reduction in the relative viability of the strains being recorded after 8 h of culture with foams containing 10 wt.% honey [54].

In vitro cytotoxicity testing clearly demonstrated the non-cytotoxic character of V5 and M5 honey cement extracts and supported the proliferation of differentiated MSCs in the extracts. In addition, ALP activity and calcium deposit production by MSCs were higher in the honey extracts than in the TTCPM extract. The enhanced differentiation of MSCs into osteoblasts was verified by analyzing the osteogenic gene expression of the MSC´s cultured in the honey cement extracts obtained after cement soaking in osteogenic medium, with an overexpression of the OP and OCN genes being observed after 15 days of culture and an overexpression of ON in the V25 extract after 7 days of culture. It was found that osteonectin is produced by active osteoblasts in order to support hydroxyapatite binding to collagens [55]. On the other hand, the OP enhances matrix mineralization and the appropriate osteointegration of new bone tissue with mature bone and promotes osteoclasts anchoring to the mineral matrix of bones during biocement resorption [56,57]. Osteocalcin is the most abundant non-collagenous protein in bone and induces the parallel growth of hydroxyapatite to the collagen fibrils, which is an important parameter for bone strength [58]. Regarding the osteogenic properties of honey, it was shown that the flavones and flavonoids in honey have good osteogenic potential, promoting the osteogenic differentiation of mesenchymal stem cells and accelerating bone fracture healing [59,60]. Therefore, the higher in vitro osteogenic activity of cells cultured in V25 or M25 extracts indicates the positive and synergistic effect of honey in conjunction with CPC, whereby the obtained composite system could be a promising therapeutic candidate for the repair of bone defects. Note that very little differences in properties were found between the V and M cements, and the statistically significant differences were only revealed in the expression of osteogenic genes (osteonectin, osteopontin, and osteocalcin), with higher levels for the V cement compared to the M cement, which could be related to the increased cytotoxicity of manuka honey.

## 5. Conclusions

New biocements based on calcium phosphate/monetite powder mixture with the addition of honey were prepared, and the influence of honey on the resulting properties of the cement was evaluated. It was shown that the setting process of cements was not affected by the addition of a liquid component containing honey in a non-cytotoxic concentration (up to 10% (*w*/*v*)) and that the setting time of ~4 min corresponded to the fast setting CPC. The original calcium phosphate cement powder mixture was completely transformed into calcium-deficient nanohydroxyapatite after 24 h of hardening in SBF. A higher fraction of long needle-like nanohydroxyapatite particles with columnar growth around the original TTCP phase particles was identified in the honey cements. The compressive strength of honey cements was reduced with the content of honey in the cement. The antibacterial activity of honey cements on *E. Coli* did not differ from the antibacterial activity of honey solutions or TTCPM cement, which may be related to the release of calcium hydroxide during cement transformation, but very low antibacterial activity was found for *S. aureus* for all cements. It was shown that the antioxidant inhibitory activity of the honey in the cement extracts correlated with the amount of released polyphenols and was close to that of common monofloral or heterofloral honeys. In vitro MTS cytotoxicity testing identified the non-cytotoxic nature of V5 and M5 honey cement extracts as well as an increase in ALP activity and calcium deposit production by the MSCs in the honey extracts. Our analysis of the osteogenic gene expression of MSCs cultured in honey cement extracts confirmed the overexpression of OP, OCN, and ON genes. In future, we will work intensively on confirming in vitro experiments on animal in vivo models; our preliminary analyses indicate the positive effect of honey in concert with biocement on the healing of bone defects.

## Figures and Tables

**Table 1:** Forward (F) and reverse (R) primers of genes used for RT-PCR determination.

Genes	Primers	References
B-actin rat	F: GTAGCCATCCAGGCTGTGTT R: CCCTCATAGATGGGCAGAGT	[25]
Type I collagen rat	F: CCAGCTGACCTTCCTGCGCC R: CGGTGTGACTCGTGCAGCCA	[26]
Osteocalcin rat	F: ACAGACAAGTCCCACACAGCAACT R: CCTGCTTGGACATGAAGGCTTTGT	[27]
Osteopontin rat	F: CCGATGAATCTGATGAGTCCTT R: TCCAGCTGACTTGACTCATG	[28]
Osteonectin rat	F: GGAAGCTGCAGAAGAGATGG R: TGCACACCTTTTCAAACTCG	[28]
Alkaline Phosphatase rat	F: AACCTGACTGACCCTTCCCTCT R: TCAATCCTGCCTCCTTCCACTA	[29]

**Table 2:** Contents of the main polyphenols and flavonoids in the honeys (determined via HPLC method).

Polyphenols	Chestnut [µg/g of Honey]	Manuka [µg/g of Honey]
Luteolin	8.2 ± 1	3.9 ± 1
Chrysin	55.6 ± 6	26.1 ± 3
Gallic acid	44.8 ± 7	46.4 ± 6
Kaempferol	18.1 ± 2	6.9 ± 1
3,4,5-trimethoxybenzoic acid	26.3 ± 3	10.4 ± 1
Chlorogenic acid	162.1 ± 11	ND
Rutin	27.5 ± 5	ND
Quercetin	7.5 ± 1	4.3 ± 1
Ferulic acid	26.8 ± 3	12.9 ± 2
Coumaric acid	76.0 ± 6	32.4 ± 2
Syringic acid	37.9 ± 5	16.6 ± 3
Methylsyringate	37.0 ± 4	134.3 ± 12
Caffeic acid	68.2 ± 8	31.8 ± 4
Protocatechuic acid	36.7 ± 3	100.3 ± 11
**Sugars**	**[wt%]**	**[wt%]**
Fructose	50.1 ± 2	40.5 ± 2
Glucose	27.5 ± 1	34.0 ± 2
Saccharose	3.8 ± 0.5	2.3 ± 0.3
Maltose	5.7 ± 0.8	4.8 ± 0.8

## Data Availability

Data will be made available upon request.
